# Respiratory syncytial virus infection of microglia exacerbates SH-SY5Y neuronal cell injury by inducing the secretion of inflammatory cytokines: A Transwell *in vitro* study

**DOI:** 10.22038/ijbms.2020.49193.11263

**Published:** 2021-02

**Authors:** Xiao-Yan Zhang, Xiao-Cheng Zhang, Hai-Yang Yu, Yun Wang, Jason Chen, Yang Wang, Li Yu, Guo-Xin Zhu, Xiu-Jing Cao, Sheng-Hai Huang

**Affiliations:** 1 Department of Microbiology, School of Basic Medical Sciences, Anhui Medical University, Hefei, Anhui Province 230032, P.R.China; 2 Anhui No.2 Provincial People’s Hospital, Hefei, Anhui Province 230000, P.R.China; 3 Department of Pathology and Cell Biology, Columbia University, New York, NY 10032, United States; 4 Department of Pediatrics, First Affiliated Hospital of Anhui Medical University, Hefei, Anhui Province 230032, P.R.China; 5 Department of Epidemiology and Health Statistics, School of Public Health, Anhui Medical University, Hefei, Anhui Province 230032, P.R.China; 6 School of Life Sciences, Anhui Medical University, Hefei, Anhui Province 230032, P.R.China

**Keywords:** Cytokines, In vitro techniques, Microglia, Neurons, Respiratory syncytial virus - infections

## Abstract

**Objective(s):**

To elucidate the mechanism of Respiratory Syncytial Virus (RSV) infection and central neuronal disease and to understand the role of microglia in neuronal injuries during RSV infection.

**Materials and Methods:**

The effects of RSV and the cytokines produced by RSV-infected CHME-5 microglial cells on SY5Y neuronal cells were evaluated based on an* in vitro* Transwell coculture system. Five treatment groups were established in this study, including the normal control SY5Y group, RSV+SY5Y infection group, (cytokine+CHME-5)+SY5Y Transwell group, (RSV+CHME-5)+SY5Y Transwell group, and (RSV+cytokine+CHME-5)+SY5Y Transwell group. The morphological and physical alterations in SY5Y cells and their synapses were analyzed by confocal microscopy. The mRNA and protein expression levels of TLR3/RIG-I, as well as the expression of Hv1, in microglia were measured by qRT-PCR and Western blot assays. In addition, the apoptosis ratio of neuronal cells was determined by flow cytometry.

**Results:**

RSV infection activated the protein expression of Hv1 protein in microglia *in vitro* (*P*<0.05), induced morphological changes in SY5Y cells, lengthened synapses (73.36±0.12 μm vs 38.10±0.11 μm), simultaneously activated TLR3 and RIG-I protein expression (*P*<0.05), upregulated the secretion of the inflammatory cytokines TNF-α, IL-6, and IL-8 (*P*<0.01), and increased the apoptosis rate of SY5Y cells (*P*<0.01).

**Conclusion:**

The results demonstrate that RSV infection of microglia can induce SY5Y neuronal cell injury and stimulate apoptosis through inflammatory cytokine release.

## Introduction

Respiratory syncytial virus (RSV) is a member of the *Orthopneumovirus* genus in the *Pneumoviridae *family (1). RSV is an encapsulated, nonsegmented single-stranded negative-strand RNA virus that is thought to cause severe illness and is one of the most common pathogens associated with respiratory viral diseases in young children worldwide (2-4). Although most of the RSV infection-associated diseases are related to airway inflammation, such as bronchiolitis and pneumonia (5), clinical studies have shown that approximately 2% of RSV-infected patients develop central nervous system (CNS) symptoms (6), such as epilepsy, central apnea, lethargy, feeding difficulty or dysphagia, abnormal muscle tone or strabismus, cerebrospinal fluid (CSF) abnormalities and encephalopathy, and there have been an increasing number of reports of RSV-related encephalopathy (7, 8).

Inflammatory factors induced in the CNS by RSV infection have been associated with pathological alterations in neurons (9-11). Long-lasting insufficient perfusion in the brain may be involved in RSV-induced encephalopathy (12). This condition remains to be fully characterized but may include unilateral hemisphere lesions.

The CNS has a complex blood-brain barrier to protect the brain from infection by most pathogens. The barrier, however, may be disrupted in inflammation to permit small molecules, including toxins, antibiotics, and viruses, to translocate from the blood to the brain. More recently, cytokines have been shown to be very important for the development of acute encephalopathy (3). The overexpression of inflammatory cytokines in the body or brain may induce mitochondrial and vascular endothelial cell disorders and apoptosis, which is thought to be the cause of acute encephalopathy (13). After RSV infection, the activation of microglia, the production of inflammatory cytokines and oxidized compounds, and the secretion of neurotransmitters may cause cognitive impairment (14). In addition, RSV infection may also lead to the occurrence of encephalopathy and systemic inflammatory response syndrome (SIRS), which is substantially related to the excessive generation of pro-inflammatory cytokines (e.g., interleukin-1β (IL-1β), interleukin-6 (IL-6), and tumor necrosis factor alpha (TNF-α)). In fact, it has been reported that RSV infection increased the level of IL-6 in spinal fluid and serum, as well as the level of interleukin-8 (IL-8) in serum (15), suggesting that cytokine storms might be associated with the pathogenesis of RSV encephalopathy. However, the pathophysiology of RSV encephalopathy has yet to be elucidated.

Microglial cells are innate immune cells that reside in human brain tissue and have long been considered to be closely related to the pathogenesis of neurodegenerative diseases. There is increasing evidence that activated microglial cells may be a chronic source of a variety of neurotoxic factors leading to progressive neuronal injuries, including IL-1, TNF-α, nitric oxide (NO), and reactive oxygen species (ROS) (16). Microglial cells can be chronically activated by exposure to a single stimulus (such as lipopolysaccharide) or multiple stimuli for continuous stimulation, leading to the loss of neurons over time (17). With the deepening of research, ROS and reactive microgliosis (the response of microglia to neuronal injuries) have been considered critical potential mechanisms for the activation of chronic and neurotoxic microglial cells, especially under the conditions that lead to Parkinson’s disease (18).

CNS diseases are commonly accompanied by the activation of astrocytes and glial cells (19). Hepatitis C virus (HCV) infection may lead to viral replication in glial cells and astrocytes in the brain, which could be related to chronic fatigue and cognitive disorders (20). In an *in vitro* study involving human herpes simplex virus 1 (HSV-1) infection, glial cells were found to be involved in the immune response in the CNS, and TLR2 in glia mediated oxidative injury in nerves (21, 22). HIV may quickly enter the brain and persistently infect macrophages and glial cells. These cells subsequently release soluble factors that disturb calcium stability and induce oxidative stress, resulting in nerve injury (14). Furthermore, glial cells may be infected and activated by Chandipura virus (CHPV) and subsequently release pro-inflammatory factors to promote neurodegeneration. In chronic infection, microglial cells could be the major source of inflammatory mediators. Cytotoxins released from activated glial cells may induce neuronal death, suggesting that the activation of glia plays an essential role in the pathogenesis of CHPV encephalitis (23). These studies suggested that the activation of microglial cells is a significant feature in neuronal inflammation in the CNS; therefore, the inhibition of this activation may be beneficial in the management of some neuronal infections and autoimmune diseases (24).

It was previously demonstrated that RSV could infect microglia and Neuro-2a (N2a) mouse neuroblastoma cells *in vitro*, and the expression of Toll-like receptor 3 (TLR3)/Toll-like receptor 7 (TLR7) in RSV-infected N2a cells was time-dependent (25). However, reports on the relationship between microglia and neuronal cells are very limited (26), and the mechanism of neuronal injury in the context of RSV infection remains unclear (27-29). In the current study, the effects of RSV infection and cytokines on microglial cells were investigated. The morphological and physical alterations in neurons and synapses were analyzed. The mRNA and protein expression levels of TLR3/retinoic acid-inducible gene I (RIG-I) and the protein changes in human voltage-gated proton channel 1 (HVCN1, Hv1) in microglia were measured. The findings of this study help to further understand the role of activated microglia in neuronal injury in the context of RSV infection.

## Materials and Methods

***Cells, virus, and reagents***

Human neuroblastoma SH-SY5Y cells (ATCC®CRL-2266, ATCC, Manassas, VA, USA) were kindly provided by Professor Yu-Xian Shen (Anhui Medical University, Hefei, Anhui, P.R. China) and were cultured using a 1:1 ratio blend of Dulbecco’s modified Eagle’s medium (DMEM) and F12 medium, which was supplemented with 10% fetal bovine serum (FBS), 100 U/ml penicillin and 100 mg/ml streptomycin.

CHME-5 microglial cells and human laryngeal epithelial cells (HEp-2) were preserved in the laboratory at the Department of Microbiology of Anhui Medical University. The cells were cultured in DMEM medium containing 10% FBS, 100 U/ml penicillin and 100 μg/ml streptomycin. These cells were cultured separately in different incubators at 37 °C with 5% CO_2_.

The long strain of RSV was kindly provided by Professor Hai-Ming Wei (University of Science and Technology of China, Hefei, Anhui, P.R. China). The virus was grown in a monolayer of HEp-2 cells and was harvested when the cytopathic effect (CPE) reached > 90%. The long strain of RSV was stored in liquid nitrogen (-196 °C) and amplified in HEp-2 cells. RSV pools were purified by polyethylene glycol (PEG) precipitation, followed by 35% to 65% discontinuous sucrose density centrifugation as described by Wong *et al *(4). The viral titer was determined to be approximately 2.5×10^8 ^PFU/ml by the Reed-Muench method. In this study, IL-1, IL-6, IL-8, TNF-α, interferon-alpha (IFN-α), granulocyte-macrophage colony-stimulating factor (GM-CSF) and other possible cytokine contaminants were not detected in the pool of virus preparations (30, 31). In addition, the limpet hemocyanin agglutination test did not detect LPS in these RSV preparations.

***Experimental design and sample collection***

SY5Y cells were divided into 5 groups as follows: (1) Normal control group: untreated normal SY5Y cells; (2) RSV+SY5Y infection group: SY5Y cells infected with RSV; (3) (cytokine+CHME-5)+SY5Y Transwell group: 0.5 ng/ml cytokine was added to CHME-5 cells cultured in the upper layer of a Transwell chamber and then applied to the lower layer of SY5Y cells; (4) (RSV+CHME-5)+SY5Y Transwell group: RSV at a TCID_50_ of 20 was added to CHME-5 cells cultured in the upper layer of a Transwell chamber and applied to the lower layer of SY5Y cells; (5) (RSV + cytokine + CHME-5) + SY5Y Transwell group: RSV at a TCID_50_ of 20 and 0.5 ng/ml cytokine were simultaneously added to CHME-5 cells cultured in the upper layer of a Transwell chamber and then applied to the lower layer of SY5Y cells.

At 12 hr, 24 hr, 48 hr, 72 hr and 96 hr post infection (pi), SY5Y cells were collected and assessed by confocal microscopy, RT-qPCR, Western blotting and flow cytometry. Cell culture supernatants were used to measure the expression of cytokines. Three replicate wells were tested in each group.

***Transwell*** ^™^ ***assay***

**Figure 1.**
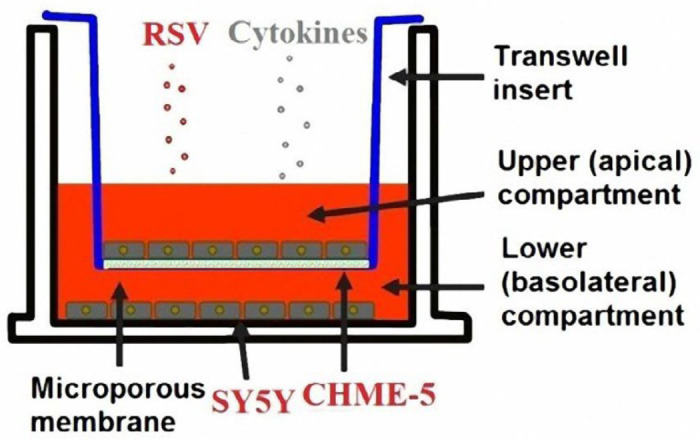
Transwell *in vitro* coculture model used in this study

To determine whether microglial cells release cytokines to affect neuronal cells, CHME-5 microglial cells were cultured in the upper Transwell chamber (Millipore, Billerica, MA, USA), and SY5Y neuronal cells were cultured in the lower Transwell chamber in a 12-well microculture plate (Figure 1) (32). Since a Transwell product with a pore diameter of less than 0.4 μm was not available on the market, a polycarbonate hydrophilic film with 0.1 μm pores (Whatman, Beijing, P.R. China) was placed on the Transwell membrane. CHME-5 cells were inoculated at a density of 5.0×10^5^/ml. SY5Y neuronal cells were seeded on the lower chamber at the same cell density. When CHME-5 cells in the upper chamber reached 80% confluence, RSV at a TCID_50_ of 20 was placed into the upper chamber for infection in the indicated groups. A mixture of cytokines each containing 0.5 ng/ml IL-6, 0.5 ng/ml IL-8, and 0.5 ng/ml TNF-α was added to the upper chamber to stimulate CHME-5 cells to release molecules that might affect SY5Y neuronal cells. To avoid cross-contamination between the apical and basolateral fluid in the Transwell insert, a strong adhesive was gently used to seal the upper Transwell chamber edge.

***ELISA analysis of TNF-α, IL-6, and IL-8***

The concentrations of TNF-α, IL-6, and IL-8 in the culture medium of the lower Transwell chamber in each group were measured by commercial ELISA kits according to the manufacturer’s instructions (Hermes Criterion Biotech, Vancouver, Canada).

***Confocal microscopy assay***

The SY5Y cell lesions and their morphological changes were visualized by confocal microscopy (33). First, several glass coverslips coated with poly-D-lysine were placed in wells of a 6-well plate (Corning Inc, New York, NY, USA). Then, the cells were added to the wells at a density of 5×10^5^ cells/well and cultured until the cells adhered to the slips. After 24 hr, the culture was inoculated with RSV at the indicated times and then fixed at room temperature with 4% formaldehyde for 30 min. Next, 0.1% Triton X-100 was added to permeabilize the cells for 20 min. Then, 10% BSA was added and incubated with the cells for 30 min to block the nonspecific binding sites. The cells were incubated overnight with primary anti-synapsin monoclonal antibodies (1:300 dilution, sc-398849, Santa Cruz Biotech, CA, USA) at 4 °C. After the cells were repeatedly washed with PBS (5 min × 3), the cells were incubated with Alexa Fluor 647-conjugated goat anti-mouse IgG secondary antibodies (1:300 dilution, Beyotime Biotech, Haimen, Jiangsu, P.R. China) for 1 hr at room temperature. Finally, the cells were incubated with DAPI (1:1000, Beyotime, Haimen, Jiangsu, P.R. China) for 10 min at room temperature and then mounted and examined under a laser confocal microscope (SP5-DM26000, Leica, Germany).

***Real-time qRT-PCR (SYBR Green)***

TLR3 and RIG-I gene transcription was assessed using real-time qRT-PCR (34). Briefly, total RNA was extracted from the SY5Y cell samples using TRI-Reagent. Then, the RNA was reverse transcribed into first-strand cDNA using a commercial reverse-transcription experimental toolkit (Takara. CN, Dalian, P.R. China). The target genes were then amplified and measured using real-time quantitative PCR. GAPDH was used as an internal control. The formula 2^−ΔΔCt^ was used to calculate the fold change in the copy number of each gene (Before using the 2^–ΔΔCt^ method, the amplification efficiencies of the target gene and the reference gene were verified. When the amplification efficiencies of the target gene and the reference gene were close to 100% and the mutual efficiency deviation was within 5%, the calculation can be performed: ΔCt(test) = Ct(target, test) – Ct(ref, test); ΔCt(calibrator) = Ct(target, calibrator) – Ct(ref, calibrator);ΔΔCt = ΔCt(test) – ΔCt(calibrator); 2^–ΔΔCt^ = the ratio of expression quantity. All primer sequences of the target genes used for qRT-PCR are listed in Table 1. The parameters of the PCR program were set to 95 °C for 5 min, followed by 40 cycles of 95 °C for 10 sec and 60 °C for 30 sec. Real-time qRT-PCR analyses were performed using a LightCycler 480 II Cycler with Roche LightCycler 480 Basic Software (Roche Applied Science, version SW 1.5.1).

***Western blot analysis***

The protein expression levels of TLR3, RIG-I, and Hv1 in each group of experiments were measured using Western blot (WB) analysis (35). First, the cells in each group were washed with PBS and lysed in lysis buffer containing phenylmethylsulfonyl fluoride (PMSF). After centrifugation, equal amounts of protein supernatant were loaded on SDS-PAGE gels and separated before being transferred to a polyvinylidene fluoride (PVDF) membrane (sc-296042, Santa Cruz Biotechnology, Santa Cruz, USA). The membrane was blocked with Tris-buffered salt solution containing nonfat milk and 0.5% Tween 20 (TBST) for 2 hr and then incubated for 12 hr at room temperature with the indicated primary antibodies: monoclonal anti-RIG-I (sc-376845 mouse IgG1 κ, Santa Cruz Biotech, Santa Cruz, CA, USA), anti-TLR3 (sc-32232 mouse IgG1 κ, Santa Cruz Biotech, Santa Cruz, CA, USA), and rabbit polyclonal anti-Hv1 (LS-C675877, LifeSpan BioSciences, Seattle, WA, USA). The dilutions for the TLR3, RIG-I, and Hv1 antibodies were 1: 500, and the dilution for the β-actin antibody was 1:1000. The membranes were washed with PBS and further incubated for 2 hr with the horseradish peroxidase (HRP)-conjugated goat anti-mouse (sc-2005, Santa Cruz Biotech, Santa Cruz, CA, USA) or goat anti-rabbit (sc-2004, Santa Cruz Biotech, Santa Cruz, CA, USA) secondary antibodies (1:10000 dilution), and the blots were developed with the enhanced chemiluminescence (ECL) kit (SuperSignal West Femto Substrate Kit, Thermo Scientific, USA). The bands were analyzed, and the relative intensities of each protein were normalized to β-actin and quantified by ImageJ ver. 1.51 software (NIH, Bethesda, MD, USA).

***Flow cytometric analysis***

To investigate SY5Y neuronal cell apoptosis, flow cytometry with Annexin V-FITC and PI staining (ab14085, Abcam. CN, Shanghai, P.R. China) was conducted according to the manufacturer’s instructions. (36). First, the cell culture mixtures were centrifuged for 5 min at 300 g at 2-8 °C to collect the suspended cells. Then, the adherent cells were digested by pancreatic enzymes without EDTA. Next, the collected cells were gently washed twice with cold PBS and centrifuged at 2-8 °C at 300 g for 5 min. The pellets were resuspended in Annexin V Reagent binding buffer to a density of 1×10^6 ^cells/ml. Finally, the cells were stained with 10 μl of PI and 5 μl of Annexin V-FITC in the dark for 15 min at 2-8 °C before being loaded on the flow cytometer for detection.

***Statistical analysis***

The calculated values are presented as the mean ±SEM. Statistical differences between the groups were determined using SPSS 19.0 software (SPSS, Chicago, IL, USA). When the variables were normally distributed, one-way analysis of variance (ANOVA) (with Tukey’s post hoc test) was conducted. Judging by the magnitude of the *P*-value, *P*<0.05 represented a significant difference, *P*<0.01 represented a highly significant difference, and *P*<0.001 represented an extremely significant difference.

## Results

***Cytokines are small enough to pass through the filter***

Before this study, no cells were loaded in the upper or lower chambers of the Transwell insert. A mixture of TNF-α, IL-6, and IL-8 was added to the upper Transwell chamber to a final concentration of 0.5 ng/ml for each cytokine (Figure 1). After 1 hr, the concentrations of the various cytokines in the upper and lower chambers of each well were analyzed by ELISA. The results showed that the concentrations of TNF-α, IL-6, and IL-8 in the lower Transwell chambers of the 6-well plates were 494 pg/ml, 487 pg/ml, and 493 pg/ml, respectively, indicating that the cytokines can pass through the Transwell insert filter.

***RSV-infected CHME-5 microglial cells secrete inflammatory cytokines***

**Figure 2:**
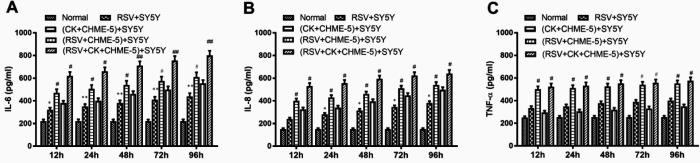
The expression of IL-6 (A), IL-8 (B) and TNF-α (C) in SY5Y cell culture supernatant at different time points was analyzed by ELISA after cytokine treatment or RSV infection of CHME-5 cells in the Transwell insert. CK indicates cytokines. The data are expressed as the average±SEM and represent the results from three independent experiments. *P<0.05, **P<0.01 compared to the normal control group. #*P*<0.05, ##*P*<0.01, ###*P*<0.001 compared to the RSV+SY5Y infection group

To investigate whether RSV infection can induce the secretion and expression of inflammatory cytokines, a mixture of inflammatory cytokines, RSV, or a combination of RSV and cytokines was added to the upper Transwell chamber, which contained a monolayer of CHME-5 cells. The concentration of each cytokine in the culture supernatant in the lower chamber of the Transwell insert, which contained SY5Y cells, was measured at the indicated time. As shown in Figure 2, compared with that of the RSV infection group, the expression of TNF-α, IL-6, and IL-8 in the RSV+ cytokine group increased significantly (*P*<0.01). These observations indicate that cytokine treatment or RSV infection induces the secretion of inflammatory cytokines by CHME-5 cells, and if these two treatments are combined, more cytokines are released into the lower chamber of the Transwell insert, which contained SY5Y cells.

***Morphological changes in SY5Y cells after RSV infection and/or cytokine treatment***

Because morphological changes in SY5Y cells were observed in pilot experiments, quantitative evaluation of axon elongation in SY5Y cells was performed by confocal microscopy at 48 and 72 hr after RSV infection or cytokine treatment. Normal SY5Y cells were homogeneous with intact and regular nuclei and an average axon length of 15.30±0.12 μm. In contrast, the RSV-infected SY5Y cells appeared to have enlarged cell bodies, irregular nuclei, and extended axons with an average length of 28.90±0.11 μm at 48 hr pi.

**Figure 3:**
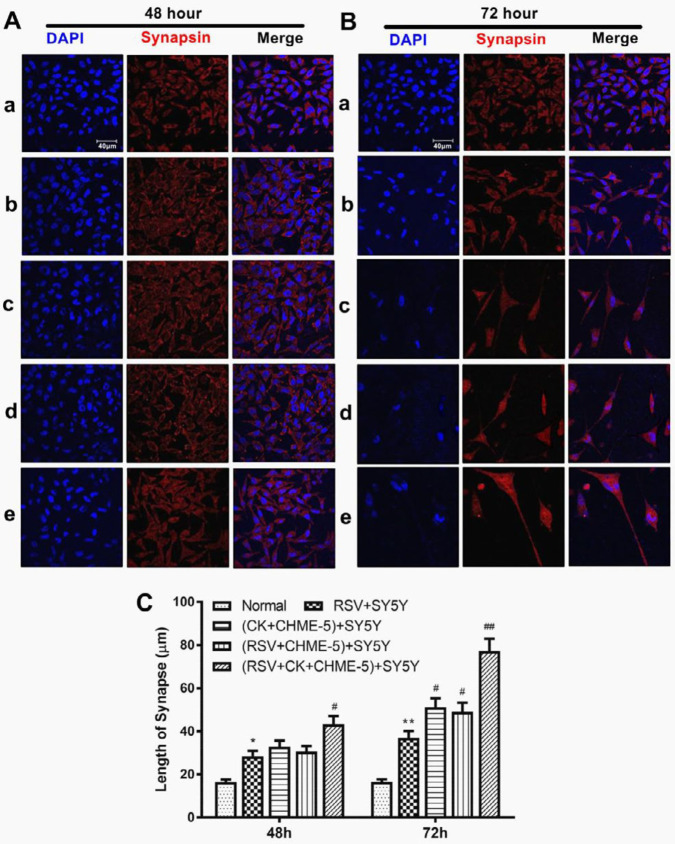
The morphological changes of SY5Y cells were analyzed by confocal microscopy after RSV infection and/or cytokine treatment. A. RSV-infected SY5Y cells at 48 hr. B: RSV-infected SY5Y cells at 72 hr. Panel a. Normal control SY5Y group. Panel b. RSV+SY5Y infection group. Panel c. (cytokine+CHME-5)+SY5Y Transwell group. Panel d. (RSV+CHME-5)+SY5Y Transwell group. Panel e. (RSV+Cytokine+CHME-5)+SY5Y Transwell group. CK indicates cytokines. Blue fluorescence shows the DAPI staining of SY5Y cell nuclei, red fluorescence represents the synapse staining of SY5Y cells, and the merged column represents the overlap of the blue and red images. C. Statistical analysis of axon elongation after RSV infection and/or cytokine treatment. The data were tested independently three times. *P<0.05, **P<0.01; #*P*<0.05, ##*P*<0.01 were all compared to the normal control group

RSV infection of SY5Y cells induced cell detachment or cell fusion, with irregular nuclei. Surviving cells appeared to have elongated axons (38.10±0.11 μm on average) at 72 hr pi. Compared with the RSV infection group, the cytokines+CHME-5 Transwell group and RSV+CHME-5 Transwell group showed more dead cells and longer synapses between the surviving cells (*P*<0.05). Furthermore, the RSV+Cytokines+CHME-5 Transwell group showed increased cell death, and the cell axons extended to 73.36±0.12 μm on average (*P*<0.01). In general, the lengths of all axons at 72 hr pi were longer than those at 48 hr pi. These observations suggest that RSV infection or cytokine treatment stimulates axon elongation in SY5Y cells (Figure 3).

***The mRNA expression of TLR3 and RIG-I increased after RSV infection and/or cytokine treatment***

After treatment with RSV and/or cytokines, the mRNA expression of TLR3 in the different groups at different time points was quantitatively measured by real-time qRT-PCR.

**Figure 4.**
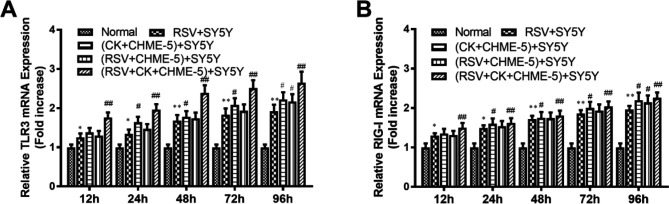
TLR3 (A) and RIG-I (B) mRNA expression in SY5Y cells at different time points in each group was measured by real-time qRT-PCR. The data are expressed as the average±SEM and represent the results from three independent experiments. *P<0.05, **P<0.01 compared to the normal control group. #*P*<0.05, ##*P*<0.01 compared to the RSV+SY5Y infection group

As shown in Figure 4A, the TLR3 mRNA level in normal cells was low and was increased after RSV infection. The difference was statistically significant after 12 hr pi (*P*<0.05). Compared to that of the RSV-infected group, TLR3 mRNA expression in the cytokine+CHME-5 Transwell group, RSV+CHME-5 Transwell group, and RSV+cytokine+CHME-5 Transwell group (*P*<0.01) was increased. These results suggest that RSV and cytokines may activate TLR3 mRNA expression in SY5Y neuronal cells.

The changing pattern of RIG-I mRNA expression in SY5Y cells after RSV infection or cytokine treatment was also similar to that of TLR3 mRNA. As shown in Figure 4B, RIG-I mRNA expression in the normal cell group was minimal and increased gradually over time after RSV infection. The difference was statistically significant starting at 12 hr pi (*P*<0.05). This result suggested that both RSV and cytokines enhance RIG-I mRNA expression in SY5Y neuronal cells (Figure 4).

***The protein expression levels of TLR3 and RIG-I in SY5Y cells, as well as the protein expression of Hv1 in CHME-5 cells, were enhanced after RSV infection and/or cytokine treatment***

**Figure 5:**
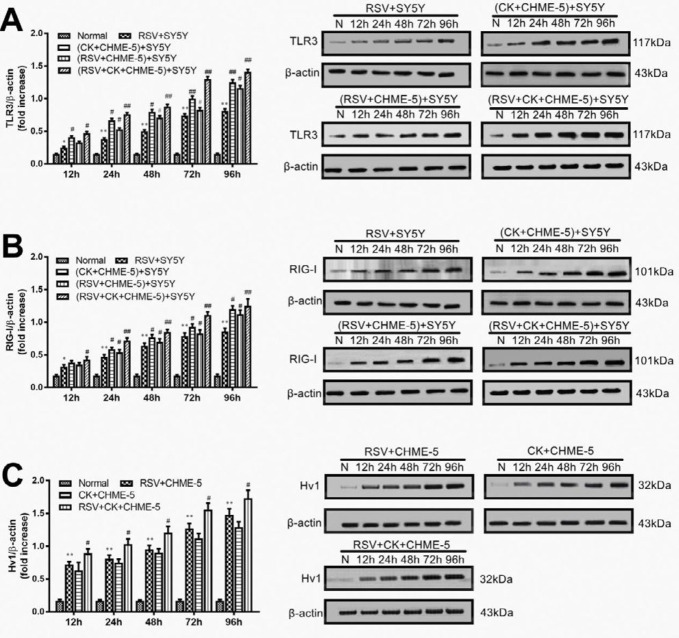
The protein expression of TLR3 (A) and RIG-I (B) in SY5Y cells and Hv1 (C) in CHME-5 cells at various time points in each group were measured by Western blot assays. The data are expressed as the average±SEM and represent the results from three independent experiments. *P<0.05, **P<0.01 compared to the normal control group. #*P*<0.05, ##*P*<0.01 compared to the RSV+SY5Y infection group

The protein expression levels were analyzed by Western blot assays. As shown in Figure 5A, TLR3 protein expression was lowest in normal cells but increased in a time-dependent manner beginning at 12 hr after RSV infection. In addition, TLR3 expression was elevated in the cytokine+CHME-5 Transwell and RSV+CHME-5 Transwell groups and was further elevated in the RSV+cytokine+CHME-5 Transwell group. These data suggested that both RSV and cytokines could activate TLR3 protein expression in SY5Y neuronal cells.

The changing pattern of RIG-I protein expression in SY5Y cells was very similar to that of TLR3 (Figure 5B). When SY5Y cells were treated with RSV or cytokines, the amount of RIG-I protein expression increased in a time-dependent manner. Significant differences appeared at 12 hr pi between normal SY5Y cells and virus-infected or cytokine-treated cells or both in cells treated with both RSV and cytokines.

Hv1 expression in microglia is necessary for ROS production, and it is responsible for neuronal death and ischemic brain damage. Hv1 protein expression in CHME-5 cells in the upper Transwell chamber was also analyzed by Western blot assays. The results showed that Hv1 expression was low in normal CHME-5 cells, but it was elevated after RSV infection or cytokine treatment and was further increased when CHME-5 cells were treated with both RSV and cytokines (Figure 5C). The increase was time-dependent, and the difference was significant at 12 hr pi.

Because of the difference in molecular weights of TLR3 and RIG-I proteins, in the same group of experiments (e.g., RSV+SY5Y and CK+CHME-5+SY5Y groups), the two proteins were separated on a single SDS-PAGE gel, and so the internal control band of β-actin used was also on the same SDS-PAGE gel; therefore, the β-actin bands in Figure 5A and Figure 5B are exactly the same.

***SY5Y cell apoptosis was increased after RSV infection and/or cytokine treatment***

**Figure 6:**
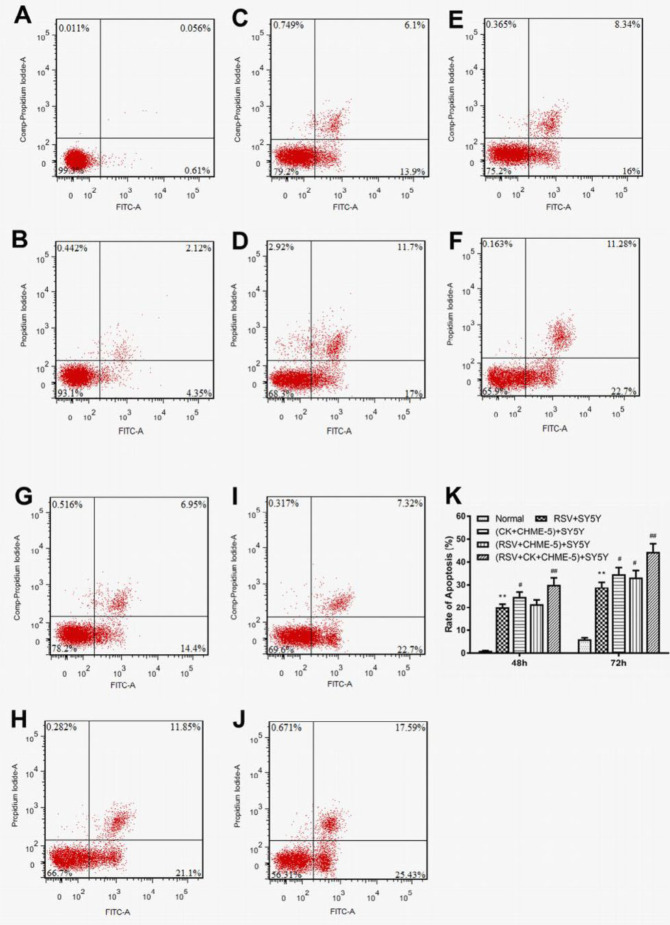
Detection of the apoptosis ratio of SY5Y cells in each group using flow cytometry. Panels A and B show the apoptotic status of SY5Y cells in the normal control group at 48 hr and 72 hr, respectively. Panels C and D show the apoptotic status of SY5Y cells in the RSV+SY5Y infection group at 48 hr and 72 hr, respectively. Panels E and F show the apoptotic status of SY5Y cells in the (cytokine+CHME-5)+SY5Y Transwell group at 48 hr and 72 hr, respectively. Panels G and H show the apoptotic status of SY5Y cells in the (RSV+CHME-5)+SY5Y Transwell group at 48 h and 72 hr, respectively. Panels I and J show the apoptotic status of SY5Y cells in the (RSV+cytokine+CHME-5)+SY5Y Transwell group at 48 hr and 72 hr, respectively. Panel K shows the quantification of the apoptotic ratio of SY5Y neuronal cells in each group. CK indicates cytokines. The data are expressed as the average±SEM and represent the results from three independent experiments. *P<0.05, **P<0.01 compared to the normal control group. #*P*<0.05, ##*P*<0.01 compared to the RSV+SY5Y infection group

The flow cytometry results showed that SY5Y cells in the normal group had reduced apoptosis (0.67%±0.01%). After RSV and cytokines were added to each group for 48 hr, the apoptosis of the RSV-infected group increased to 20.00%±0.02%, and the difference was statistically significant. (*P*<0.01). The apoptotic rate of the cytokine+CHME-5 Transwell group was 24.34%±0.01%, which was significantly higher than that of the RSV infection group. (*P*<0.05). However, the apoptotic rate in the RSV+CHME-5 Transwell group did not change significantly compared with that of the RSV-infected group. The apoptotic rate of the RSV+cytokine+CHME-5 Transwell group was 30.32%±0.01%, which was significantly higher than that of the RSV infection group. (*P*<0.01) (Figure 6).

At 72 hr after infection, apoptosis in each group was more pronounced than at 48 hr (Figure 6). Compared to that of the normal group, the apoptosis rate of the RSV infection group increased to 28.70±0.03%, which was significantly higher than that of the normal group (*P*<0.01). Apoptosis was more significant in the cytokines+CHME-5 Transwell group and the RSV+CHME-5 Transwell group than in the RSV-infection group, and the apoptosis rates were 33.98%±0.01% and 32.95%±0.02%, respectively. The differences in apoptosis between these two groups was statistically significant (*P*<0.05). The RSV+Cytokines+CHME-5 Transwell group had more pronounced apoptosis than the RSV-infected group, in which the apoptosis rate was 43.02%± 0.01%. The difference between these two groups was highly significant (*P*<0.01) (Figure 6).

## Discussion

Influenza virus may lead to encephalitis and encephalopathy by activating glial cells to release potential cytotoxic substances, such as TNF-α, IL-1β, NO, and oxygen-free radicals. These factors may trigger apoptosis. In addition, the activation and apoptosis of glial cells are crucial for the development of encephalopathy (37). In a pilot experiment performed by our group, it was found that RSV infection produced CPE in the neuronal cell line SY5Y after 48 hr, which prompted us to analyze the cellular pathogenesis and apoptosis of SY5Y cells induced by RSV and cytokines by confocal microscopy and flow cytometry. Since the Transwell cells are not centrifuged, the added cytokines may not thoroughly pass through the polycarbonate membrane in the Transwell insert. However, because that experiment was only performed to prove that overexpression can lead to reduced neuronal damage, calculating the precise levels of cytokines was not necessary and the concentration only needed to reach a level that could cause neuronal injury.

The Toll-like receptor (TLR) family is a class of evolutionarily conserved pattern recognition receptors (PRRs). These type I transmembrane proteins are natural immune receptors against infections from various pathogenic microorganisms (38). TLRs play an important role in CNS damage. When RSV infects central nervous system cells, TLR4 in the cell membrane can recognize RSV and mediate endocytosis by membrane fusion, permitting intracellular viral replication. This recognition by TLR4 may activate the natural immune response that could lead to the occurrence of neuronal damage. RIG-I is a receptor for the intracellular identification of the double-stranded RNA genome of the virus. RSV may initiate host antiviral responses through RIG-I, TLR3, TLR7, and other molecular recognition receptors. In addition, it has been found that RSV infection can induce the upregulation of TLR7 and RIG-I (39). Therefore, in this study, changes in the mRNA and protein expression of TLR3 and RIG-I were investigated.

Hv1 is a recently cloned voltage-gated proton channel that is selectively expressed in microglia but not neurons (40). The Hv1 pathway is involved in ROS production in microglia in the brain, which then leads to neuronal cell death and ischemic brain injury (41). The expression of Hv1 in microglia is necessary for the production of ROS (42). The massive production of ROS in microglia upon activation may lead to an increase in pro-inflammatory cytokines in microglia and result in neuronal death. Additionally, neuronal death and brain injury could be prevented in Hv1-knockout mice. (40, 43). The present study showed that RSV infection could induce an increase in Hv1 expression in SY5Y cells in Transwells (Figure 4C), which suggested the involvement of the Hv1 pathway in the process of viral infection in neurons. The Hv1 pathway was recently recognized as a therapeutic target for a variety of diseases, such as multiple sclerosis (44). In addition, increasing evidence has shown that microglia act as a bridge between the CNS and the immune system in autoimmune neuroinflammation (39, 45). In future experiments, we will attempt to interfere with Hv1 expression in RSV-infected glia to explore its effect on the production of ROS and pro-inflammatory cytokines.

The synapse is the structure between neurons or between neuron and the target effector cells and transmits electrical or chemical signals. Upon stimulation, neurons can produce a large number of neurotransmitters. To adapt to changes in the quantity of neurotransmitters, the length of the synaptic area increases, which may play a role in maintaining synapse structure and protect neurons from excitotoxic damage. When the brain is injured, the number of neuronal synapses increases significantly, and ischemic stimulation increases the active area of the synapse (46). Studies have shown that cytokines released from microglia, such as TNF-α and IL-10, induce neuronal synapse formation. The higher the dose of IL-10, the longer the synapse (47). Knocking out IL-10 can reduce the formation of synapses. Therefore, the effects of inflammatory cytokines induced by RSV on the formation of synapses were examined.

In the current study, the results revealed that RSV infection or exogenous cytokine treatment might induce neuronal cell apoptosis, and the surviving neurons in culture exhibited axon extension and enlargement of the cell body. When RSV-infected neurons were treated with both exogenous cytokines and cytokines that were released from glial cells in the Transwell, increased axon extension was observed (Figure 3). This might be the result of the increased culture surface for the surviving cells due to apoptosis and cell death in RSV-infected SY5Y cells.

RSV infection triggers signal transduction in infected cells and induces the synthesis and release of inflammatory cytokines to fight against viral infection, as well as the enhancement of adaptive immune responses (48). We found that both RSV and a cytokine mixture consisting of TNF-α, IL-6, and IL-8 could stimulate the gene expression of TLR3 and RIG-I, and TLR3 expression was more elevated than that of RIG-I after 12 hr (25). These observations were consistent with a report that RIG-I expression was induced in RSV-infected A549 cells in the early phase of infection, and TLR3 expression was elevated in the late phase of the infection (49).

It was previously shown that TLR3 and RIG-I might induce cellular apoptosis (50, 51). In Junin virus-infected A549 cells and Vero cells, apoptosis was increased significantly after 4 h, along with the increased expression of TLR3 and RIG-I (52, 53). Additionally, a previous study performed by our group demonstrated that the expression of TNF-α, IL-6, and IL-8 and the apoptotic rate of infected cells increased after RSV infection in the N2a neuronal cell line (25). Therefore, cytokines may play an important role in inflammation that results in neuronal apoptosis.

**Table 1:** Primer sequences for real-time qRT-PCR assay

Target gene	Sequence (5′→3′)
TLR3	F: 5’- GATCTGTCTCATAATGGCTTG-3’
	R: 5’- GACAGATTCCGAATGCTTGTG-3’
RIG-I	F: 5’- AAGAGCCAGAGTGTCAGAATC-3’
	R: 5’- AGCTCCAGTTGGTAATTTCTTGG-3’
GAPDH	F: 5’- ATCAAGAAGGTGGTGAAGCA-3’
	R: 5’- AAGGTGGAAGAGTGGGAGTTG-3’

qRT-PCR: quantitative reverse-transcription polymerase chain reaction; TLR3: toll-like receptor 3; RIG-I: Retinoic acid-inducible gene I; GAPDH: glyceraldehyde-3-phosphate dehydrogenase

## Conclusion

In general, the findings of this study are as follows: 1. RSV can infect and elongate the axons of SY5Y neuronal cells. 2. RSV may infect microglial cells, and microglia produce cytokines such as TNF-α, IL-6, and IL-8 that could injure SY5Y neuronal cells. 3. The overexpression of inflammatory cytokines can also induce the apoptosis of SY5Y neuronal cells. These observations suggest that RSV may injure neurons directly, but RSV can also infect microglial cells and induce cytokine expression that injures neuronal cells. These results indicate that the inflammatory cytokines released by RSV-infected microglia can promote neuronal cell injury. This study provided evidence for the role that microglia play in RSV-induced neuronal injury, which may provide a therapeutic target for the treatment of RSV-related encephalopathy.

## Compliance with Ethical Standards

This article does not contain any studies with human participants or animals performed by any of the authors.
